# Disinfectants and Deodorizers

**Published:** 1873-04

**Authors:** 


### DISINFECTANTS AND DEODORIZERS.

A DISINFECTANT takes away the injurious quality of a bad atmosphere. A deodorizer simply takes away the ill smell, and even that only for a time. The old-time plan was to burn sugar or tar in the sick-room. That was neither a disinfectant nor deodorizer; it only gave a more powerful "smell;" all the ill qualities of the air of the sick chamber were in it still, with that of the tar or sugar superadded.

Carbolic acid not only destroys the bad smell, but it prevents the generation of more; it disinfects, it takes away the power of further decay; it takes away all death-giving properties already existing, and prevents the formation of others, according to our latest light on the subject.

Powdered charcoal absorbs putrid gases, and condenses them, thus taking away all odor, but in time it becomes saturated, and will do no further good. Spread over tainted meat, it sweetens it, but then it must be cooked, or the taint will go on.

Some freshly slackened, sprinkled over damp places, as in cellars, sinks and gutters, is the cheapest and most easily applied for removing ill odors, and purifies the air.

A pound of copperas, costing ten cents, dissolved in a gallon or two of water in a pail, is instantaneously efficient for privies and the like.

